# Graph neural networks for multi-scale gene-drug interaction modeling in cancer: applications, challenges, and therapeutic opportunities for breast cancer drug resistance

**DOI:** 10.3389/fonc.2026.1929697

**Published:** 2026-08-25

**Authors:** Priya Rani Das, Md. Jarifur Rahman, Sowhanur Rahman Nirob, Md. Owafeeuzzaman Patwary, Md. Reazul Islam, Md. Shabiul Islam, Nibras Ahmed, Firoz Ahmed

**Affiliations:** 1 Department of Computer Science, American International University-Bangladesh (AIUB), Dhaka, Bangladesh; 2 Department of Computer Science, University at Albany, State University of New York (SUNY), Albany, NY, United States; 3 Centre for Advanced Devices and Systems, Centre of Excellence for Robotics and Sensing Technologies, Multimedia University, Persiaran Multimedia, Cyberjaya, Selangor, Malaysia; 4 Faculty of Artificial Intelligence and Engineering, Multimedia University, Persiaran Multimedia, Cyberjaya, Selangor, Malaysia; 5 Department of Computer Science, Rajshahi University of Science and Technology, Rajshahi, Bangladesh

**Keywords:** breast cancer, drug resistance, drug-target interaction, explainable artificial intelligence, gene-drug interactions, graph neural networks, multi-omics integration, precision oncology

## Abstract

Breast cancer drug resistance remains a major clinical challenge driven by complex genetic, signaling, and microenvironmental interactions. Conventional machine learning and deep learning represent genes, drugs, and patients as independent feature vectors, limiting their ability to capture biological relationships governing therapeutic response. Graph neural networks have emerged as a powerful paradigm by modelling biological systems as interconnected networks rather than isolated entities. This review synthesizes recent advances in graph neural network based multi-scale modelling of gene-drug interactions across cancer research, emphasizing translational relevance to breast cancer drug resistance. Although many architectures originate from pan-cancer or methodological studies, their potential for breast cancer is critically assessed while distinguishing models directly validated from those requiring adaptation. Recent architectures, including hierarchical, heterogeneous, knowledge graph-guided, explainable, and graph-augmented models, demonstrate strong predictive performance across oncology tasks. These studies computationally nominated potential targets such as FAK, FLT3, COX8A, SEC61G, and CYP27B1, though predictions require experimental validation in breast cancer resistance models. The field has leveraged established synthetic lethal relationships, such as BRCA1/PARP, as benchmarks for GNN-based discovery frameworks. Despite encouraging progress, current evidence remains largely retrospective, benchmark-based, or preclinical. Cross-cohort heterogeneity, limited interpretability, and scarce breast cancer-specific resistance validation represent central limitations. Future integration with spatial transcriptomics, multimodal omics, and federated learning may improve precision oncology, but rigorous biological validation and interdisciplinary collaboration are essential for clinical implementation.

## 1 Introduction

Breast cancer remains the most common malignancy among women worldwide. Targeted and endocrine therapies have substantially improved patient outcomes, yet intrinsic and acquired drug resistance persists as a major clinical challenge. This resistance hinders long-term success across all breast cancer subtypes, including hormone receptor-positive and triple-negative disease Gao et al. (1) Ran et al. (2). Drug resistance in breast cancer arises through numerous highly interconnected mechanisms, including mutations in drug-binding receptors, activation of compensatory signaling pathways, drug efflux via transporters, and remodeling of the tumor microenvironment. These processes operate across multiple biological scales, spanning gene–drug interactions, pathway-level circuitry, and cellular network dynamics Zhou and Zhou (3)Alpsoy and Sezerman (4). Understanding and predicting these multi-scale interactions is therefore central to identifying durable therapeutic targets and guiding precision oncology strategies for breast cancer patients.

Conventional computational approaches, including single-omics machine learning models and standard deep learning frameworks, have advanced drug response prediction but typically represent genes, drugs, and patients as independent feature vectors, discarding the relational and topological context that governs resistance biology Zack et al. (5). Multi-omics integration strategies have improved predictive accuracy, yet most still struggle with high dimensionality relative to limited sample sizes, poor cross-cohort generalizability, and an inability to capture hierarchical gene–pathway–drug dependencies simultaneously Yan et al. (6)Li et al. (7). Critically, these models often function as opaque predictors, offering little mechanistic insight into how specific gene–drug interactions drive resistance, which restricts their translational utility for therapeutic target discovery.

Although several reviews have examined graph neural networks (GNNs) in drug discovery Zhang and Liu (8)Wang et al. (9), cancer research Chen et al. (10), or single-cell omics independently, a comprehensive synthesis focused specifically on GNN-driven multi-scale gene-drug interaction modeling with relevance to breast cancer drug resistance remains lacking. It is important to note that while many GNN architectures discussed in this review were developed on pan-cancer datasets or methodological benchmarks, we explicitly evaluate their translational potential for breast cancer research and distinguish between breast cancer-validated models, pan-cancer models with breast cancer relevance, and methodological frameworks requiring further adaptation. Existing reviews rarely integrate molecular resistance mechanisms, multiomics data fusion strategies, explainable graph learning architectures, and therapeutic target discovery within a unified systems biology framework Hsu et al. (11). Consequently, important opportunities for connecting computational advances with clinically actionable insights remain insufficiently explored.

This review addresses this gap by systematically synthesizing recent advances in GNN-based approaches for modeling breast cancer drug resistance. We critically distinguish between models that have been directly validated on breast cancer data and those that provide methodological frameworks with high translational potential for breast cancer research. This distinction is essential for accurately representing the current state of the field and identifying priority areas for future experimental validation. Specifically, this review aims: (i) to examine the molecular mechanisms underlying breast cancer drug resistance from a systems biology perspective; (ii) to categorize emerging GNN architectures, including hierarchical, heterogeneous, knowledge graph-guided, explainable, and graph-augmented language model frameworks; (iii) to evaluate strategies for integrating multi-omics and single-cell data into graph-based learning pipelines; and (iv) to discuss current challenges, translational opportunities, and future directions for therapeutic target discovery and precision oncology.

Building on this synthesis, we provide an architecturally structured analysis of GNN-driven multi-scale gene–drug interaction modeling in breast cancer. By natively encoding biological systems as graphstructured data, GNNs capture relational gene–drug–pathway dependencies that traditional feature-based models fail to represent. This framework integrates multi-omics and single-cell data with advanced graph learning paradigms, offering a biologically grounded and clinically oriented perspective on overcoming drug resistance and accelerating therapeutic discovery.

## 2 Methodology of literature selection

This mini-review followed a structured narrative literature search guided by the PRISMA 2020 reporting framework to ensure a transparent and reproducible study selection process. Literature was retrieved from four major scientific databases: PubMed, Scopus, IEEE Xplore, and Web of Science. The final search was conducted on June 15, 2026. Publications from January 2024 to June 2026 were considered to capture recent advances in multi-omics integration, gene-drug interaction modeling, and resistance-aware predictive frameworks for breast cancer.

### 2.1 Search strategy

The search strategy combined controlled vocabulary and free-text keywords using Boolean operators (AND, OR). Core search terms included *“graph neural network,” “GNN,” “breast cancer,” “drug resistance,” “gene-drug interaction,” “drug-target interaction,” “multi-omics,” “single-cell,” “heterogeneous graph,” “knowledge graph,” “synthetic lethality,”* and *“therapeutic target.”*.

To ensure comprehensive retrieval, database-specific syntax adaptations were applied as follows:

- PubMed: MeSH terms were used where applicable (e.g., “Breast Neoplasms”[MeSH], “Drug Resistance, Neoplasm”[MeSH]).
- Scopus: TITLE ABS KEY field restrictions were applied.
- IEEE Xplore: “Abstract only” and “Full Text & Metadata” search fields were used.
- Web of Science: Topic search (TS) was applied.

The following Boolean search strings served as the foundation and were adapted per platform:

- Primary Search String:
  (“graph neural network” OR “GNN” OR “graph convolutional network” OR “graph attention network” OR “heterogeneous graph” OR “knowledge graph”) AND (“breast cancer” OR “breast neoplasms” OR “mammary carcinoma”) AND (“drug resistance” OR “drug tolerance” OR “treatment resistance” OR “therapy resistance” OR “chemoresistance”) AND (“gene-drug interaction” OR “drug-target interaction” OR “gene-drug association” OR “multi-omics” OR “transcriptomics” OR “genomics”).
- Secondary Search String (Broader Scope).
  (“graph neural network” OR “GNN”) AND (“cancer” OR “tumor” OR “neoplasm”) AND (“drug response” OR “drug sensitivity” OR “drug resistance”) AND (“gene expression” OR “mutation” OR “copy number” OR “methylation”).
- Synthetic Lethality and Target Discovery Search String.
  (“graph neural network” OR “GNN”) AND (“synthetic lethality” OR “therapeutic target” OR “biomarker discovery”) AND (“breast cancer” OR “pan-cancer”) AND (“gene interaction” OR “protein-protein interaction”).

The initial search identified 88 records. After removing duplicate publications, 78 records remained for title and abstract screening. Following title and abstract screening, 22 records were excluded, leaving 56 records for full-text assessment. Following detailed eligibility evaluation, 33 records were excluded, resulting in 23 studies that satisfied all predefined inclusion criteria and were included in this mini-review.

Title/abstract screening and full-text eligibility assessment were conducted independently by three reviewers. Any disagreements were resolved through discussion and consensus. When consensus could not be reached, final decisions were made by the senior authors according to the predefined eligibility criteria.

**Figure 1:**
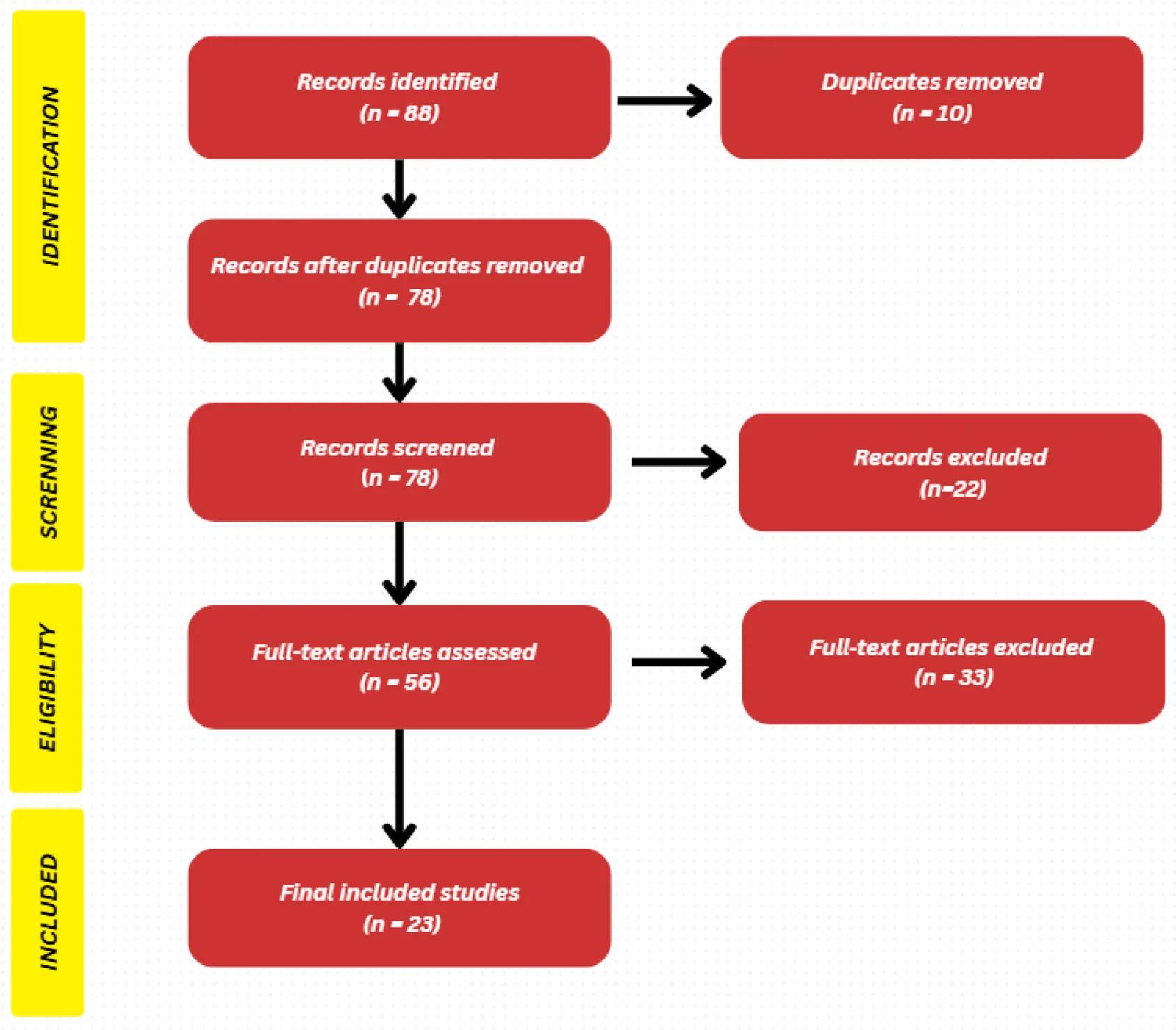
PRISMA 2020 flow diagram of study selection.

Eligible studies were evaluated for methodological quality, scientific relevance, and clinical significance. Particular emphasis was placed on studies investigating GNN-based modeling of breast cancer drug resistance, multi-omics data integration, drug-target interactions, synthetic lethality prediction, biomarker discovery, and interpretable graph-based learning frameworks. Studies focused solely on general cancer prognosis without explicit drug resistance relevance, methodological development without breast cancer application, or non-graph-based approaches were excluded. The complete literature selection workflow is illustrated in **Figure 1**.

### 2.2 Inclusion criteria

- Articles published between January 2024 and June 2026.
- Peer-reviewed original research articles, systematic reviews, and high-quality review papers.
- Studies focusing on GNN-based modeling of cancer drug response, resistance mechanisms, or genedrug interactions.
- Studies incorporating multi-omics data (genomics, transcriptomics, epigenomics, proteomics) into graph-based frameworks.
- Studies incorporating single-cell transcriptomics or spatial transcriptomics data into graph-based frameworks for resistance modeling.
- Research involving drug-target interaction prediction, synthetic lethality discovery, or therapeutic target identification.
- Studies employing heterogeneous graph architectures, knowledge graph-guided models, or explainable graph neural networks.
- Studies with demonstrated or high translational potential for breast cancer research.
- Articles published in English.

• Studies providing sufficient methodological detail on graph construction, model architecture, or validation approach.

### 2.3 Exclusion criteria

- Articles published before January 2024.
- Studies without clear relevance to breast cancer research or cancer drug resistance mechanisms.
- Research based exclusively on traditional machine learning or standard deep learning without graphbased architectures.
- Studies addressing only general cancer prognosis, survival prediction, or diagnosis without explicit relevance to drug resistance or therapeutic response.
- Studies describing only methodological development without biological or clinical application, unless the methodology has clear translational potential for breast cancer.
- Pan-cancer studies that do not include breast cancer cell lines, breast cancer data, or demonstrate clear translational relevance to breast cancer.
- Conference abstracts, editorials, letters, perspectives, commentaries, and opinion articles.
- Duplicate publications or substantially overlapping reports.
- Studies with insufficient methodological detail to assess graph construction, model architecture, or validation approach.
- Non-English publications.

### 2.4 Classification of GNN models by breast cancer validation status

To provide clarity regarding the translational relevance of reviewed models, we classify them into three categories based on their validation status:

Category 1: Breast Cancer Validated.

- Jassim et al. (12): Validated in mouse models of triple-negative breast cancer
- Lee et al. (13): Validated on retrospective breast cancer clinical cohorts
- Yan et al. (6): Validated on TCGA-BRCA breast cancer dataset
- Zhang and Liu (8): Applied directly to breast cancer protein communities

Category 2: Pan-Cancer Validated with Breast Cancer Relevance.

- Zhang et al. (14) - GraphTCDR: Includes breast cancer cell lines in training
- Chen et al. (10) - DGIB4SL: Identified BRCA1/PARP, directly relevant to breast cancer therapy
- Liu et al. (15) - DrugFormer: Analyzed drug-resistant cancer cells, with potential breast cancer application

Category 3: Methodological Frameworks (No Breast Cancer Validation).

- Zhao et al. (16) - EviDTI: Methodological framework requiring breast cancer adaptation
- Jing et al. (17) - H2GnnDTI: General DTI prediction framework
- Wang et al. (9) - XGDP: General drug sensitivity prediction framework

This classification is essential for accurately interpreting the current evidence base and identifying priority areas for future experimental validation in breast cancer models.

## 3 Molecular mechanisms of drug resistance in breast cancer

Drug resistance remains one of the principal barriers to successful breast cancer treatment and arises through complex interactions among genomic, transcriptomic, epigenetic, metabolic, and microenvironmental factors. The underlying mechanisms differ across molecular subtypes and therapeutic modalities, reflecting the heterogeneity of breast cancer and the dynamic evolution of tumor cells during treatment. Increasing evidence from multi omics studies demonstrates that resistance is driven by interconnected biological networks rather than isolated molecular alterations, highlighting the need for systems level approaches capable of integrating diverse molecular data Ran et al. (2) Hsu et al. (11).

Hormone receptor-positive breast cancer accounts for approximately 70% of all breast cancer cases and is primarily treated with endocrine therapies, including selective estrogen receptor modulators, selective estrogen receptor degraders, and aromatase inhibitors. Despite their clinical benefit, nearly half of patients eventually exhibit either intrinsic or acquired resistance Gao et al. (1) Ran et al. (2). Activating mutations in the estrogen receptor, particularly D538G and Y537S, reduce sensitivity to endocrine therapies by promoting ligand-independent receptor activation. In addition, approximately 30% of patients receiving aromatase inhibitors acquire *ESR1* mutations accompanied by hyperactivation of signaling pathways involving PI3K, AKT, mTOR, Notch, NF-*κ*B, FGFR, and the IRE1-XBP1 pathway. These coordinated molecular alterations promote estrogen-independent proliferation and disease progression while substantially reducing therapeutic efficacy Gao et al. (1).

Resistance to HER2 targeted therapies represents another major clinical challenge. Although agents targeting HER2 have significantly improved outcomes in HER2 positive breast cancer, resistance commonly develops through activation of compensatory signaling pathways, including PI3K, AKT, and mTOR signaling, loss of PTEN, altered HER2 receptor expression, epithelial mesenchymal transition, and remodeling of the tumor microenvironment Gao et al. (1) Ran et al. (2). These mechanisms involve extensive interactions among receptors, signaling proteins, stromal cells, and immune components, emphasizing the importance of network based computational models for understanding treatment failure.

Cyclin dependent kinase 4 and 6 inhibitors have become standard therapy for hormone receptor positive metastatic breast cancer. However, resistance frequently develops through RB1 loss, cyclin E amplification, CDK2 activation, fibroblast growth factor receptor signaling, and activation of the PI3K, AKT, and mTOR pathway, allowing tumor cells to bypass cell cycle arrest Gao et al. (1)Ran et al. (2). Likewise, resistance to inhibitors targeting the PI3K, AKT, and mTOR pathway arises through feedback activation of receptor tyrosine kinases, PTEN loss, compensatory MAPK signaling, metabolic adaptation, and intratumoral heterogeneity, illustrating the complexity of pathway crosstalk in therapeutic resistance Gao et al. (1) Ran et al. (2).

Targeted therapies exploiting defects in DNA damage repair have also transformed breast cancer treatment. Poly ADP ribose polymerase inhibitors demonstrate substantial efficacy in patients with BRCA1 or BRCA2 mutations, yet resistance may develop through restoration of homologous recombination repair, BRCA reversion mutations, replication fork stabilization, and altered DNA damage response pathways Ran et al. (2). Similarly, antibody drug conjugates have emerged as effective therapies for HER2 positive and HER2 low breast cancer, although reduced antigen expression, impaired intracellular trafficking, lysosomal dysfunction, activation of drug efflux transporters, and resistance to the cytotoxic payload can limit long term therapeutic benefit Ran et al. (2).

Triple negative breast cancer demonstrates distinct mechanisms of resistance because of its molecular heterogeneity and lack of established therapeutic targets. Increased expression of ATP binding cassette transporters reduces intracellular accumulation of chemotherapeutic agents, while activation of cancer associated fibroblasts, M2 macrophages, immune checkpoint pathways, and programmed death ligand 1 promotes an immunosuppressive tumor microenvironment that supports treatment resistance Zhou and Zhou (3) Chen et al. (10). Additional mechanisms, including enhanced DNA repair, epithelial mesenchymal transition, cancer stem cell enrichment, and metabolic reprogramming, further contribute to resistance against conventional chemotherapy Zhou and Zhou (3) Chen et al. (10).

Recent multi omics studies further demonstrate that drug resistance is a dynamic and continuously evolving process. Transfer learning based integration of genomic, transcriptomic, and proteomic data has linked LDHB mediated metabolic reprogramming and PINK1 dependent mitophagy with therapeutic resistance across multiple cancer types Alpsoy and Sezerman (4). Single cell transcriptomic trajectory analysis has identified RPS6KB1 as a key regulator distinguishing drug sensitive from resistant cellular states during breast cancer progression Iida and Okada (18). Furthermore, context dependent gene interaction networks have revealed novel therapeutic vulnerabilities capable of mitigating treatment resistance by identifying dynamic changes in gene dependencies Jassim et al. (12). Collectively, these findings indicate that resistance emerges from interconnected molecular networks spanning genes, signaling pathways, cellular states, and the tumor microenvironment rather than from isolated genetic alterations.

**Figure 2:**
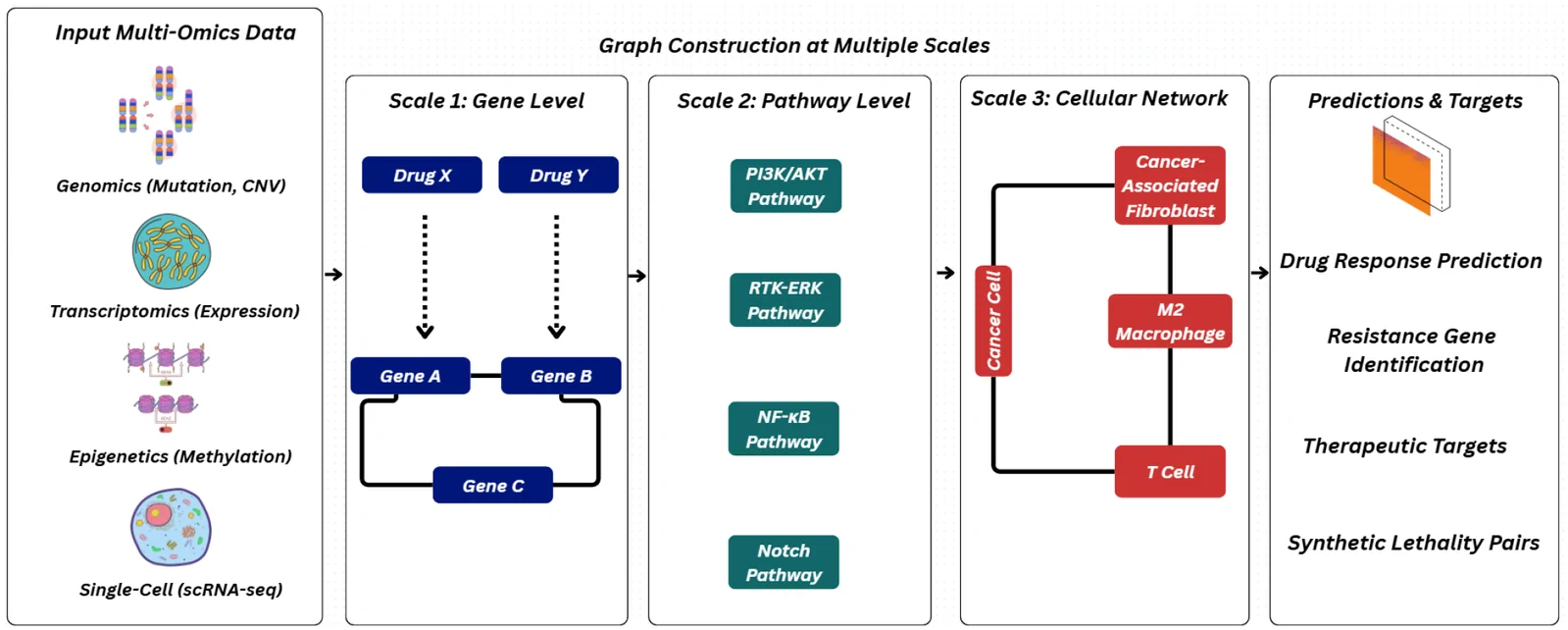
Multi-scale biological graph from genes to pathways to tumor microenvironment, enabling GNN-based prediction of drug response, resistance genes, therapeutic targets, and synthetic lethality in breast cancer.

The network driven nature of breast cancer drug resistance provides a strong biological rationale for graph based artificial intelligence approaches. Graph neural networks can naturally represent interactions among genes, proteins, signaling pathways, cells, and drug molecules while integrating heterogeneous multi omics data within a unified framework. Such models have demonstrated promise for predicting drug response, identifying resistance associated biomarkers, prioritizing therapeutic targets, and discovering synthetic lethal interactions in precision oncology Yan et al. (6) Li et al. (7) Zhang et al. (14). **Figure 2** illustrates how multi scale biological graphs spanning genes, pathways, and the tumor microenvironment support graph neural network based prediction of drug response, resistance genes, therapeutic targets, and synthetic lethality in breast cancer. Throughout this review, the node types represented by each GNN architecture vary depending on the biological question and data availability. Models discussed below represent nodes as drugs, target proteins, genes, cell lines, individual cells, or biological pathways. **Table 1** summarizes representative graph neural network architectures for breast cancer drug resistance modeling, whereas **Table 2** compares recent multi omics graph based approaches for cancer drug response and resistance prediction.

**Table 1:** Representative graph-based models for oncology drug response, resistance, and therapeutic target discovery with relevance to breast cancer.

Study	Disease context	Graph structure	Prediction task	Prediction target	Validation	Experimental evidence	Key limitation
8	Breast cancer	Protein–protein + GO graph	Biomarker discovery	Resistance biomarkers	Computational	Literature support only	No resistant cell validation
Jing et al. (17) (H2GnnDTI)	Pan-cancer	Drug–protein heterogeneous graph	Drug–target interaction	Drug–protein binding	Benchmark datasets	None	Not breast cancerspecific
Zhao et al. (16) (EviDTI)	Pan-cancer	Drug graph + protein graph	Drug–target interaction	Binding prediction + confidence	Benchmark datasets	Case studies	Requires protein structures
Zhang et al. (14) (GraphTCDR)	Multiple cancers	Drug–gene–cell heterogeneous graph	Drug response	IC50/sensitivity	Cross-validation	None	No external cohort validation
Lee et al. (13)	Breast cancer	Knowledge graph	Pathway interpretation	Clinical assay prediction	Retrospective cohort	Clinical datasets	No prospective validation
Yan et al. (6)	Breast cancer	Gene regulatory hierarchy	Prognosis	Tumor risk/ survival	TCGA validation	None	Not drug resistance
9(XGDP)	Pan-cancer	Drug molecular graph	Drug response	Drug sensitivity	Public datasets	None	Limited mutation modelling
Chen et al., (10)(DGIB4SL)	Pan-cancer	Gene interaction graph	Synthetic lethality	SL gene pairs	Benchmark datasets	Literature validation	Limited interpretability
Liu et al. (15) (DrugFormer)	Pan-cancer	Gene knowledge graph	Drug sensitivity	Sensitive/resistant cells	Public datasets	scRNA-seq analysis	Scalability issues
Dong et al. (19) (DKPEGraphSYN)	Pan-cancer	Drug interaction graph	Drug synergy	Combination response	Benchmark datasets	None	No breast cancer validation

**Table 2:** Multi-omics integration approaches for cancer drug response and resistance modeling.

Study	Omics layers integrated	Node types	Graph construction	Prediction task	Disease context	Performance	Limitation
GraphTCDR (Zhang et al. (14))	Gene expression, mutation, CNV	Drugs, genes, cell lines	Heterogeneous graph with omics correlation edges	Drug response (IC_50_)	Pan-cancer (PRISM; >500 cell lines, >2,000 drugs)	PCC +3.60%; SCC + 4.30%	No breast cancer-specific analysis or cross-cohort validation.
Multilevel GNN (Yan et al. (6))	Gene expression, methylation, CNV, GRNs, KEGG/Reactome	Genes, modules, pathways	Gene → module → pathway hierarchy	Tumour risk and survival prediction	TCGA-BRCA	Identified SEC61G and CYP27B1	No drug resistance endpoint; limited interpretability.
DeepCCDS ^†^ (Wu et al. (20))	Driver mutations, CNV, transcriptomics	Driver genes	Prior knowledge-guided graph	Drug sensitivity prediction	GDSC, CCLE	Improved interpretability; reduced noise	No breast cancer resistance validation.
(Li et al. (7))	scRNA-seq, spatial, epigenomics, proteomics	Cells	Similarity or spatial graph	Cell-state classification and signalling inference	General oncology	107 studies reviewed	No breast cancer-specific evaluation; scalability issues.

†The symbol indicates a graph-informed framework that is not a pure GNN.

## 4 GNN architectures for gene–drug interaction modeling

**Figure 3:**
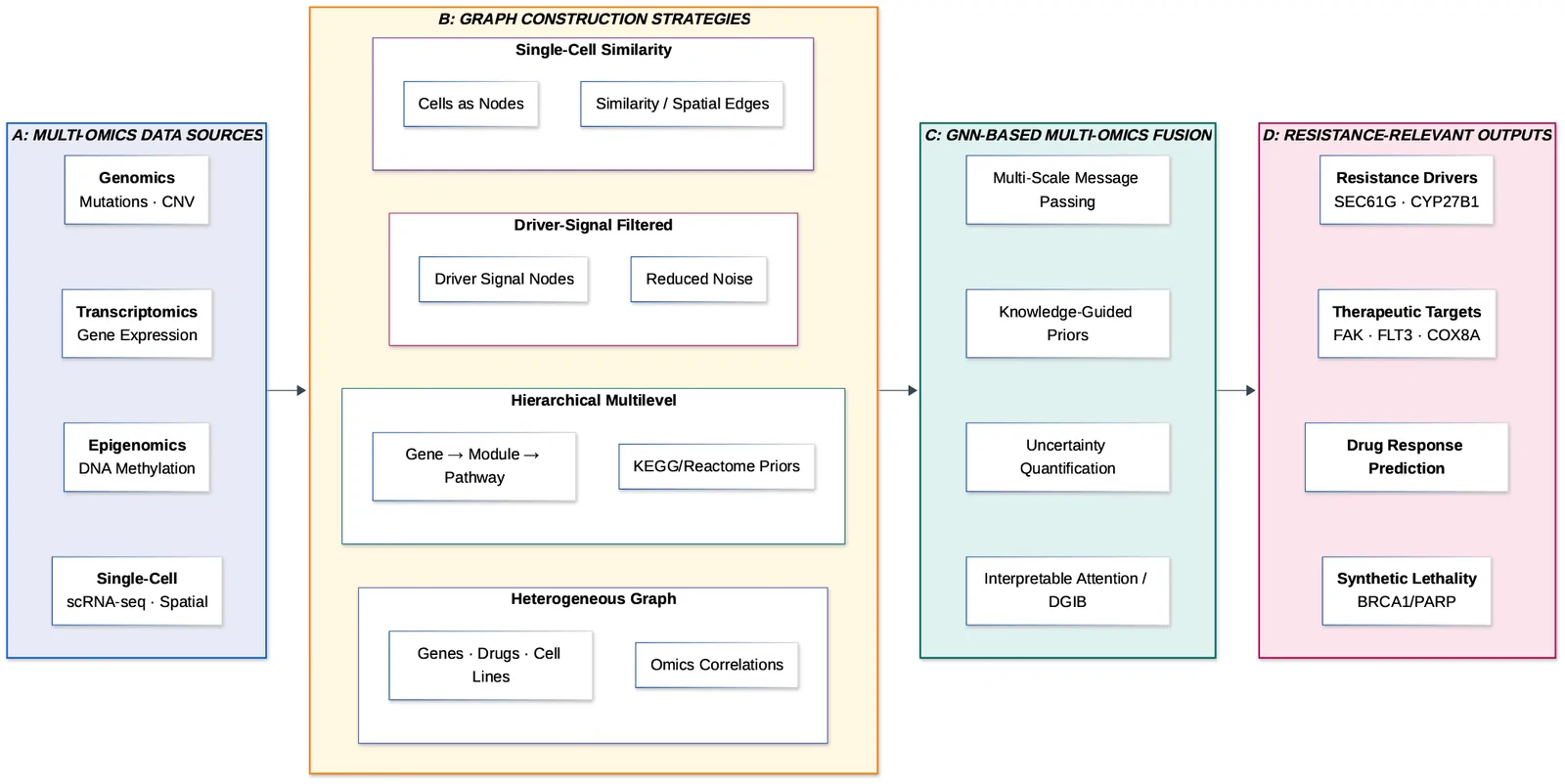
End-to-end GNN framework integrating multi-omics data **(A)**, graph construction strategies **(B)**, fusion methods **(C)**, and resistance outputs **(D)** for breast cancer drug resistance modeling.

An overview of the end-to-end GNN pipeline-from multi-omics data input and graph construction to fusion methods and resistance prediction outputs-is provided in **Figure 3**. Graph neural networks (GNNs) have emerged as the most architecturally suitable class of models for gene-drug interaction modeling, owing to their native capacity to operate on non-Euclidean, relational biological data Li et al. (7). While several of these models have been developed on pan-cancer datasets, their direct relevance to breast cancer drug resistance requires careful contextualization. The following discussion is therefore organized by distinguishing models with direct breast cancer validation from those with demonstrated potential for adaptation.

### 4.1 Hierarchical and heterogeneous models for multi-scale biology

At the node classification level, GraphX-Net applies GNN layers combined with Shapley value attribution to breast cancer gene expression graphs, achieving interpretable prediction of recurrence risk and identifying key prognostic gene nodes Basaad et al. (21). Building on this foundation, hierarchical GNN architectures extend node-level representations to multi-scale biological structures. Zhang and Liu’s hierarchical GNN constructs a multi-scale tree of breast cancer protein communities supervised by gene ontology terms, identifying SUPT6H and RAD21 as biomarkers and nominating three FDA-approved drugs-mercaptopurine, pioglitazone, and colchicine-as candidate therapeutics. However, these nominations remain to be validated in breast cancer resistance models Zhang and Liu (8).

#### 4.1.1 Methodological frameworks with high translational potential

Heterogeneous GNNs, which encode multiple node and edge types within a unified graph, have proven particularly powerful for drug–target interaction (DTI) prediction. H2GnnDTI constructs a bipartite heterogeneous graph of drugs and target proteins, applying hierarchical message passing to capture both local binding site features and global interaction topology Jing et al. (17). EviDTI advances this paradigm by incorporating evidential deep learning for uncertainty quantification, integrating drug 2D topological graphs, 3D spatial structures, and protein sequence features. Uncertainty-guided predictions computationally nominated modulators targeting tyrosine kinases FAK and FLT3, demonstrating that confidence-aware DTI modeling can prioritize high-value therapeutic targets for experimental validation Zhao et al. (16). However, these nominations remain entirely computational and have not been experimentally validated in any cancer model, including breast cancer. The methodological framework nonetheless provides a clear pathway for breast cancer-specific application, though substantial experimental validation would be required before clinical consideration.

#### 4.1.2 Breast cancer relevant models

GraphTCDR extends heterogeneous GNNs to multi-omics drug response prediction and represents a more directly applicable framework, as it encodes gene expression, somatic mutation, and copy number data to predict drug response across cell lines, including breast cancer lines in its pan-cancer training data Zhang et al. (14). However, even this model lacks breast cancer-specific validation, underscoring the translational gap that remains to be addressed.

### 4.2 Knowledge graph-guided and explainable models

Knowledge graph–guided GNNs represent the most biologically grounded architectural family, embedding curated pathway databases (KEGG, Reactome) directly into the graph learning process. Lee et al. proposed a multi-layered knowledge graph GNN specifically for breast cancer multi-gene assay interpretation, revealing through attention analysis that Oncotype DX, Prosigna, and EndoPredict are uniformly regulated by RTK-ERK-ETS-mediated proliferation signaling-a finding with direct implications for resistance bypass mechanisms Lee et al. (13). Complementarily, the prior knowledge-guided multilevel GNN of Yan et al. sequentially integrates gene regulatory networks and pathway information across omics layers, with GNN-Explainer and IGscore identifying SEC61G and CYP27B1 as non-linearly survival-associated genes Yan et al. (6). DeepCDG employs a graph convolutional network to fuse mutation, expression, and methylation data for cancer driver gene identification, though this framework has not been specifically validated for resistance prediction in breast cancer Wu et al. (20). DKPEGraphSYN applies dual kernel density estimation with positional encoding in a GNN framework to predict drug synergy combinations, offering a graph-based route to overcoming single-agent resistance Dong et al. (19).

^†^DeepCCDS is a graph-informed multi-omics framework rather than a pure GNN. A prior biological network guides feature selection before prediction using a feedforward neural network.

Explainability is a non-negotiable requirement for clinical deployment of GNN predictions. XGDP represents drugs as molecular graphs processed by a GNN module, applying deep attribution algorithms to reveal the specific functional groups and cancer-cell gene interactions underlying drug response-identifying EGFR as a key target of Afatinib through attention analysis Wang et al. (9). DGIB4SL addresses the interpretability gap in synthetic lethality prediction by introducing a diverse graph information bottleneck objective with 13 motif-based adjacency matrices, generating multiple faithful mechanistic explanations for gene pairs including clinically validated BRCA1/PARP interactions central to breast cancer therapy Chen et al. (10). Finally, DrugFormer integrates gene-based knowledge graphs with a language model architecture, leveraging scRNA-seq data from resistant cancer cells to computationally nominate COX8A as a resistance-relevant target Liu et al. (15). However, this nomination lacks functional validation; COX8A has not been experimentally demonstrated to drive resistance or to be therapeutically actionable. The study nonetheless exemplifies how graph-augmented language models can extend GNN capabilities into single-cell precision oncology and generate testable hypotheses for future validation. A comparison of multi-omics GNN approaches, including integrated omics layers, graph construction methods, and performance limitations, is provided in **Table 2**.

## 5 Multi-omics data integration strategies

Effective modeling of gene–drug interactions in breast cancer requires the concurrent integration of complementary omics layers. Genomic data-including somatic mutations and copy number aberrations (CNAs) capture the mutational landscape driving resistance, while transcriptomic profiles reflect the downstream gene expression consequences of those alterations Alpsoy and Sezerman (4). GraphTCDR encodes all three layers-gene expression, somatic mutation, and CNV as node and edge features within a heterogeneous graph, enabling cancer drug response prediction that outperforms single-omics baselines by capturing inter-omics dependencies invisible to vector-based models Zhang et al. (14). DeepCCDS employs a graph-informed feature selection strategy, using a prior knowledge network to characterize cancer driver signals before passing these features to a feedforward neural network for drug sensitivity prediction. While not a pure GNN-lacking the message-passing and relational learning core of true graph neural networks-it illustrates how prior biological knowledge can guide feature engineering in multi-omics settings Wu et al. (20).

Incorporating prior biological knowledge substantially enhances the utility of multi-omics graphs. The prior knowledge–guided multilevel GNN of Yan et al. sequentially integrates gene expression, DNA methylation, and copy number data with gene regulatory networks and KEGG/Reactome pathway priors, constructing hierarchical graph layers in which each level corresponds to a distinct biological scale-from gene to module to pathway Yan et al. (6). This architecture overcomes the curse of dimensionality inherent in high-feature, low-sample multi-omics datasets, and deepCDG demonstrates the same principle by fusing mutation, expression, and methylation into a GCN framework for cancer driver gene identification Wu et al. (20).

Single-cell transcriptomics represents the most granular integration layer, revealing cellular heterogeneity masked in bulk omics analysis. GNNs model individual cells as graph nodes, with edges defined by transcriptomic similarity or spatial proximity, enabling the simultaneous inference of cell type, cell state, and intercellular signaling across 107 reviewed applications spanning epigenomics, spatial transcriptomics, and proteomics Li et al. (7). In the context of breast cancer drug resistance, scRNA-seq pseudotime modeling of tamoxifen-treated MCF-7 cells captured five distinct drug-response subpopulations and mapped resistance-associated gene regulatory networks at single-cell resolution-a level of mechanistic specificity unattainable by bulk multi-omics integration alone Iida and Okada (18).

## 6 Therapeutic target discovery and clinical translation

The translation of GNN-based modeling into actionable therapeutic targets constitutes the ultimate objective of this computational paradigm. The RECODR framework, published in Cancer Cell (2025), exemplifies this translation most compellingly: by embedding cancer transcriptomes as graphs and measuring gene co-expression context drift during treatment, RECODR identifies resistance-driving targets and designs combination therapies validated in mouse models, while simultaneously predicting novel treatment options for patients with triple-negative breast cancer Jassim et al. (12). This marks a critical milestonedemonstrating that graph-based computational target discovery can generate experimentally and clinically validated therapeutic strategies rather than merely computational predictions.

At the molecular interaction level, DTI-oriented GNNs provide structured pipelines for computational target prioritization. EviDTI’s uncertainty-guided predictions computationally nominated tyrosine kinases FAK and FLT3 as potential targets Zhao et al. (16). Importantly, these remain computational predictions only; no experimental validation has been performed for FAK or FLT3 in breast cancer models. The framework nonetheless enables efficient experimental triage by focusing limited validation resources on confident predictions, though such validation remains to be conducted. The knowledge-guided heterogeneous GNN of (KGDRP) bridges phenotype-based and target-based drug discovery by learning from both observed drug phenotypes and known target interaction graphs, identifying candidate therapeutic targets that would be inaccessible through either approach alone Ye et al. (22). DGIB4SL advances target discovery into the synthetic lethality domain, predicting gene pairs-including BRCA1/PARP interactions central to clinical breast cancer therapy-where co-inactivation selectively kills cancer cells, with multiple faithful mechanistic explanations generated per gene pair Chen et al. (10).

Drug combination modeling represents a clinically critical extension of single-target discovery, particularly for overcoming resistance through multi-node pathway disruption. DKPEGraphSYN applies dual kernel density estimation with positional encoding to a GNN architecture, predicting synergistic drug combinations by capturing the weighted probability density of gene expression and inter-drug molecular interactions Dong et al. (19). Boolean signaling pathway modeling of breast cancer cell lines revealed that MEK and STAT3 inhibitor combinations produce only moderate synergy in MDA-MB-468 cells due to negative contributions from mTORC1 and NF-*κ*B, demonstrating the interpretable mechanistic specificity that graph-based combination models uniquely afford over black-box prediction approaches Taoma et al. (23). DrugFormer further integrates knowledge graph-guided language modeling with scRNA-seq data to computationally nominate COX8A as a potential resistance-relevant target in drug-resistant cellular subpopulations Liu et al. (15). However, this nomination lacks functional validation; COX8A has not been experimentally demonstrated to drive resistance or to be therapeutically actionable in breast cancer. The study nonetheless exemplifies how single-cell-resolved computational analysis can generate testable hypotheses and pinpoint potential vulnerabilities invisible to bulk-omics methods.

### 6.1 Distinguishing computational prediction, experimental validation, and clinical validation

It is essential to distinguish between computational predictions, experimentally validated targets, and clinically established relationships. Among the targets discussed in this review, the evidence hierarchy varies considerably:

- Computational Predictions Only: FAK, FLT3, and COX8A represent purely computational nominations. FAK and FLT3 were identified through EviDTI’s DTI framework Zhao et al. (16) but have not been experimentally validated in any cancer model, including breast cancer. COX8A was nominated through DrugFormer’s scRNA-seq analysis Liu et al. (15) but lacks functional validation demonstrating its role in resistance or therapeutic actionability.
- Survival Associations (Not Resistance): SEC61G and CYP27B1 were identified as nonlinearly survival-associated genes through multilevel GNN analysis of TCGA-BRCA data Yan et al. (6). Importantly, these associations relate to overall survival, not drug resistance specifically. The distinction between prognostic biomarkers and resistance drivers is critical and requires careful interpretation.
- Clinically Established Relationships (Not Novel GNN Discoveries): BRCA1/PARP represents a clinically validated synthetic lethal interaction that is well-established in the literature and used clinically for PARP inhibitor therapy in BRCA-mutant breast cancer. While DGIB4SL Chen et al. (10) successfully predicted this known relationship as a benchmark, the interaction itself is not a novel GNN discovery. The value of such predictions lies in demonstrating that GNNs can recover known synthetic lethal interactions, thereby supporting their potential for discovering novel relationships that warrant experimental investigation.

This classification underscores that the majority of GNN-nominated targets remain at the computational prediction stage, with limited experimental validation and virtually no clinical validation specifically for breast cancer resistance. Systematic experimental validation pipelines are therefore essential to translate computational discoveries into actionable therapeutic strategies.

### 6.2 Comparative analysis of GNN models for breast cancer drug resistance

To provide meaningful comparative insights, we present a synthesis of key GNN models with demonstrated or high potential for application to breast cancer drug resistance. **Table 3** a structured comparison of these models, outlining their biological question, key findings in the context of breast cancer, validation status, and primary limitations.

**Table 3:** Comparative analysis of Key GNN models for breast cancer drug resistance.

Model	Biological question	Key findings in breast cancer context	Validation status	Primary limitation
Zhang and Liu (8)	Biomarker & drug nomination	Identified SUPT6H, RAD21; nominated 3 repurposable drugs	Computational nomination	Not validated in resistant models
Lee et al. (13)	Clinical assay interpretation	Revealed RTK-ERK-ETS as common regulator	Retrospective cohort	Not linked to resistance
Yan et al. (6)	Prognosis & biomarker discovery	Identified SEC61G, CYP27B1 as survivalassociated	TCGA-BRCA survival analysis	Survival, not resistance
Jassim et al., (12) (RECODR)	Resistance target & combination design	Designed validated combinations for TNBC	*In vivo* mouse validation	Requires extensive resources
Zhao et al., (16) (EviDTI)	Drug-target interaction	Computationally nominated FAK, FLT3	Computational prediction only	No validation in any cancer model
Chen et al., (10) (DGIB4SL)	Synthetic lethality	Recovered known BRCA1/PARP interaction	Known clinical relationship	Not a novel GNN discovery
Liu et al., (15) (DrugFormer)	Resistance target discovery	Computationally nominated COX8A	Computational nomination only	Lacks functional validation

#### 6.2.1 Key insights from comparative analysis

This comparative analysis reveals several important patterns. First, models explicitly designed for breast cancer analysis (e.g., Yan et al. (6) Zhang and Liu (8) Lee et al. (13)) tend to produce more clinically interpretable findings but often lack experimental validation of nominated targets. Second, pan-cancer models like EviDTI (Zhao et al. (16)) and DGIB4SL (Chen et al. (10)) offer powerful methodological frameworks and have nominated targets (e.g., FAK, FLT3) or validated mechanisms (e.g., BRCA1/PARP) that are directly relevant to breast cancer, yet the majority of their predictions remain untested in breast-specific resistant models. Third, a critical gap exists between computational target nomination and experimental validation, with only RECODR (Jassim et al. (12)) providing prospective *in vivo* validation in breast cancer models.

## 7 Challenges and future directions

**Table 4** outlines the key challenges-data limitations, model generalizability, interpretability, and scalabilityalong with proposed future directions for GNN-based breast cancer drug resistance modeling. Despite their considerable promise, GNN-driven gene-drug interaction models face several unresolved methodological challenges that must be addressed before clinical deployment. Multi-omics datasets for individual cancer patients remain constrained by small sample sizes relative to feature dimensionality, predisposing models to overfitting and the curse of dimensionality Yan et al. (6). The significant variation created by data harmonization across omic modality (including batch correction, missing data imputation and cross-platform normalisation) reduces model generalisability across separate cohorts Hsu et al. (11) and is compounded by GNN models trained on established drug-target interaction (DTI) benchmarks exhibiting little generalisability to unseen, novel DTI pairs, even when uncertainty-aware architectures like EviDTI partially mitigate the model incompleteness via calibration mechanisms Zhao et al. (16). Model interpretability remains a critical bottleneck for clinical translation. Attention mechanisms widely used in KG-GNNs for synthetic lethality prediction lack fidelity-generating single, unreliable explanations per gene pair and failing to capture high-order structural dependencies within biological interaction networks Chen et al. (10). In the single-cell domain, the ultra-high dimensionality and inherent sparsity of scRNAseq data impose severe computational scalability constraints, with GNN architectures facing prohibitive memory and training costs as graph sizes expand across thousands of cells and tens of thousands of gene nodes Li et al. (7). Bridging the gap between computational target prediction and experimental validation further demands coordinated multi-disciplinary frameworks that the field has not yet standardized Hsu et al. (11).

**Table 4:** Challenges and future directions for GNN-based breast cancer drug resistance modeling.

Study	Challenge category	Specific challenge	Proposed future direction
Yan et al. (6)	Data limitations	Small sample size, high dimensionality	Federated learning for multi-institutional data
Hsu et al. (11)	Data heterogeneity	Batch effects, missing data, cross-platform normalisation	Standardised harmonisation protocols
Zhao et al. (16)	Model generalisability	Poor transfer to unseen DTI pairs	Uncertainty-aware GNNs with calibration
Chen et al. (10)	Interpretability	Attention gives single, unreliable explanations	Diverse graph information bottleneck (DGIB) with multiple motifs
Li et al. (7)	Single-cell scalability	Ultra-high dimensionality, sparsity, memory cost	Efficient GNN sampling/hierarchical pooling
Hsu et al. (11)	Translation to clinic	Gap between prediction and experimental validation	Prospective clinical validation pipelines
Li et al. (7)	Emerging opportunity	Spatial transcriptomics for tumor microenvironment resistance	Integrate spatial transcriptomics with GNNs
Hsu et al. (11)	Long-term frontier	Individualised adaptive therapy	Quantum graph optimisation/N-of1 models

Several emerging directions hold particular promise for overcoming these limitations. Federated learning architectures enable privacy-preserving multi-institutional collaboration on patient-level omics data without centralised data sharing-directly addressing both the sample size deficit and regulatory compliance barriers that impede clinical deployment Hsu et al. (11). Spatial transcriptomics, integrated with GNN frameworks, offers a route toward decoding TME-level resistance mechanisms at tissue-spatial resolution, extending beyond single-cell inference to intercellular communication networks Li et al. (7). Quantum computing–enhanced graph optimization and patient-centric N-of-1 modeling represent longer-horizon frontiers that may ultimately enable fully individualized, dynamically adaptive therapeutic strategies for breast cancer drug resistance Hsu et al. (11).

## 8 Conclusion

Graph neural networks (GNNs) have emerged as a powerful framework for modeling the complex biological networks underlying breast cancer drug resistance. By integrating multi-omics data, molecular interaction networks, and drug response information, GNN-based approaches have advanced drug-target prediction, synthetic lethality discovery, biomarker identification, and drug combination modeling, providing a systems-level perspective for therapeutic discovery.

Three key takeaways emerge from this review. First, GNNs have demonstrated considerable potential for identifying novel therapeutic targets and prioritizing precision treatment strategies. Second, despite these advances, a critical validation gap persists: most computational predictions remain hypothesisgenerating and have not been experimentally tested in resistant versus sensitive models. This gap between computational nomination and clinical actionability represents the single greatest barrier to translation. Third, significant methodological challenges including limited data availability, dataset heterogeneity, poor model generalizability to unseen data, and inadequate interpretability continue to constrain clinical adoption and must be systematically addressed through standardized benchmarking and explainable AI approaches.

Looking ahead, future research should focus on three priorities: (i) establishing standardized experimental validation pipelines for computational predictions, (ii) developing shared benchmark resources that integrate multi-omics, drug response, and resistance data, and (iii) designing clinically interpretable and generalizable GNN models capable of generating mechanistically testable hypotheses across diverse patient cohorts.

In summary, GNNs represent a promising paradigm for advancing precision oncology in breast cancer. Their long-term impact, however, will depend not on computational elegance alone, but on a collective commitment to rigorous experimental validation, reproducible benchmarking, and close integration with clinical research. While the foundational computational frameworks have matured considerably, substantial work remains to translate these approaches into clinical practice. Continued efforts to address data limitations, improve model generalizability, enhance interpretability, and bridge the validation gap will be essential to realize the full translational potential of graph-based learning for overcoming breast cancer drug resistance.
